# Physical contributors to dose in patients with dual-port temporary tissue expanders treated post-mastectomy with 10 MV x-rays: a Monte Carlo study

**DOI:** 10.1088/2057-1976/adce10

**Published:** 2025-04-25

**Authors:** Ramon Ortiz, Bruce Faddegon, Manju Sharma, José Ramos-Méndez

**Affiliations:** University of California San Francisco, Department of Radiation Oncology 1600 Divisadero Street, San Francisco, CA 94143, United States of America

**Keywords:** post-mastectomy radiotherapy, temporary tissue expanders, Monte Carlo, photoneutrons

## Abstract

***Objective***. To evaluate how radiation interactions, influenced by a dual-port temporary tissue expander (TTE), impact dosimetry in post-mastectomy radiotherapy (PMRT) with 10 MV x-rays. ***Approach***. The individual dose contributions from the radiation interaction processes within the patient, specifically the photoelectric effect, pair production, bremsstrahlung, and neutrons, were evaluated in a PMRT treatment involving the dual-port AlloX2 TTE using Monte Carlo simulations. The plan setup was two 10 MV tangential half-beam-blocked fields (40 Gy in fifteen fractions). Individual contributions of the different physical processes were computed using a dedicated physics list that allows to activate/deactivate each process. The yield of photoneutrons produced in TTE neodymium ports (ρ = 7.4 g/cm^3^) and their impact on equivalent neutron dose were computed using previously validated physics modules. The effect of the presence of the TTE was estimated by comparing results in plans with and without the TTE. ***Results***. The presence of the TTE reduced the dose to the breast skin distal to the ports up to 19.3% of the prescribed dose. The contribution of the photoelectric effect and bremsstrahlung was confined to the metallic ports, accounting for 9% and 1% of the total dose. Pair production accounted for 20% of the dose deposited within the ports and contributed 2.2 Gy and 0.9 Gy to the maximum dose to the lung and heart, respectively. We found that no photoneutron was produced in the TTE, not having an effect on the equivalent neutron dose to the patient. ***Significance***. This work extended the current knowledge on the impact of TTE on dose distributions, including neutron contamination, in PMRT treatments.

## 1. Introduction

Post-mastectomy radiotherapy (PMRT) is often used in the treatment of patients diagnosed with advanced breast cancer to control potential microscopic tumor spread to the chest wall and adjacent breast tissue (Remick & Amin 2023). At the time of treatment, some patients may have a temporary tissue expander (TTE) in place between the chest wall and the skin, used prior to the permanent breast reconstruction. TTE’s are typically made of a silicone bag filled with saline solution and include one or two magnetic ports of high atomic number (Z) to guide the injection of the filling into the implant valves. The presence of the high-Z materials used in the ports, such as titanium and neodymium, may have an impact on the dosimetry of those treatments.

Several studies have evaluated the impact of TTE on dose distributions. Beam attenuation distal to the ports of up to 30% and 16% for 6 and 10 MV beams, respectively (Thompson & Morgan 2005, Damast *et al* 2006, Chatzigiannis *et al* 2011, Dinc 2020), along with side-scattering and back-scattering (Thompson & Morgan 2005, Chatzigiannis *et al* 2011), have been reported as the main dosimetric effects in the vicinity of the ports. Despite different studies attribute those effects to distinct physical interactions of radiation with matter (e.g., secondary particles produced by pair production)(Chatzigiannis *et al* 2011), the specific processes contributing to the dose deposited within the TTE and patient tissues have not been investigated. Whereas secondary scattered electrons are not likely to have an impact on body tissues due to their short range (~5 mm), some studies argued that the beam attenuation may lead to a non-negligible underdosing at points outside the TTE (Thompson & Morgan 2005, Chatzigiannis *et al* 2011). For instance, using thermoluminescent dosimeters (TLD), Damast *et al* (Damast *et al* 2006) reported up to 22% and 14% skin underdosing due to single-port TTE’s in clinical treatments consisting of 6 MV and 15 MV tangential beams, respectively. For *in vivo* measurements in VMAT and 3D-conformal radiotherapy treatments at different energies (6, 10 and 18 MV), a 5 to 7% reduction in the dose to the skin in the shadow of the magnets was found (Gee *et al* 2016, Yoon *et al* 2018). A comparable degree of attenuation was also reported for dual-port TTEs (2–5%) irradiated with 3D-conformal partial arcs (6 MV beams) within the first centimeter posterior to the port (Ramos-Méndez *et al* 2023).

The aim of this work was to characterize the dose distributions in breast treatment plans using the AlloX2 dual-port TTE by individually evaluating the physical interaction processes of 10 MV x-ray beams. In addition, the neutron contribution produced by the interaction of such high-energy x-ray beams with the high-Z materials of the ports, which was disregarded in previous studies, is evaluated. For those purposes, a PMRT treatment consisting of two opposed tangential 10 MV beams and the dual-port AlloX2 TTE (Sientra Inc., Santa Barbara, CA, USA) were considered. The rationale behind selecting this treatment modality for the present study is that (i) the contribution of pair production and photoneutron production is expected to be non-negligible for such high-energy beams, and (ii) the dosimetric impact of the AlloX2 TTE has not been studied yet using this technique and energy.

## 2. Methods and materials

### 2.1. Clinical case

A fully anonymized clinical case from our institution undergoing a PMRT treatment was selected for this study. The treatment was delivered before breast reconstruction and included the sub-pectoral dual-port AlloX2 TTE. The treatment, planned with RayStation (version 11A), consisted of two 10 MV tangential parallel opposed half beam blocked fields. The plan and ROI structures were exported for their use in Monte Carlo simulations as described in section 2.2. The dose prescription was 40 Gy in fifteen fractions. The PTV for dose evaluation was defined as the 95% isodose line tangent to the breast, subtracting 5 mm of skin.

### 2.2. Monte carlo simulations

As reported in previous works (Thompson & Morgan 2005, Gee *et al* 2016), analytical methods used in treatment planning systems (TPS) may underestimate or overestimate the dosimetric impact of the metallic ports. Thus, in this study, Monte Carlo (MC) simulations were used. In our simulations, the patient was modeled by fully anonymized patient’s DICOM-CT images. This study was approved by the UCSF committee on human research with a waiver of informed consent and was compliant with the Health Insurance Portability and Accountability Act (HIPAA).

The TOPAS toolkit (Toolkit for Particle Simulation) version OpenTOPAS v4.0 based on Geant4.11.1 (Perl *et al* 2012, Faddegon *et al* 2020) was used. The linac model used was the TrueBeam system with the 120 millenium MLC (Varian Medical Systems, Palo Alto, CA, USA). Simulation parameter files were created with a validated TPS-TOPAS interface (Ortiz *et al* 2024). The radiation source consisted of fifty 10 MV phase space (PHSP) files generated for the TrueBeam system provided by Varian (www.myvarian.com/montecarlo). To improve simulation efficiency, the Geometrical Particle Splitting variance reduction technique implemented in TOPAS (Ramos-Méndez *et al* 2013) was used with no Russian roulette, using a split factor of 10. In addition, to improve neutron yield efficiency, the uniform split variance reduction technique with a split factor of 500 was applied within the patient volume for the calculation of neutron yields. Finally, a track-length estimator scorer was used to improve the efficiency of neutron dose calculation. The precision of the calculated total dose, averaged over voxels with more than half of the maximum dose, was 1.5%.

The method used to manage metal artifacts in CT images and delineate the port geometry has been previously described and validated in the literature (Ramos-Méndez *et al* 2023). Briefly, a rigid registration of a predefined geometry template, based on manufacturer specifications for the port’s shape, material, and density, is applied to the CT scan. The CT streak artifacts produced by the high-Z metal ports were corrected by using different contours (Region of Interest, ROI), and assigning a material to the different ROI. The ROIs and assigned materials were: a saline solution composed 0.3538% Na, 0.5435% Cl, 11.0990% H and 88.0153% O, and density of 1.0046 g/cm^3^ for the TTE filling (Redler *et al* 2018), ICRU lung and heart tissues (International Commission on Radiation Units and Measurements 1989) for these two organs, and water for the rest of patient tissue and for a 5 mm-bolus covering the patient skin. The metallic ports of the AlloX2 TTE were modelled by a titanium structure with a neodymium magnet enclosed, as detailed in a previous work (Ramos-Méndez *et al* 2023). Air material was assigned outside the patient volume.

### 2.3. Calculation of the contributions to the dose from the different physical processes

The impact of metallic ports in dose distributions was evaluated by comparing simulation results of plans with and without the ports. The description of the plan with the ports is described in detail in section 2.1. In the plan without ports, all the TTE (including the port volume) was overridden by water (see section 2.2). The same beam configuration as in the plan with ports was used. The treatment plan, not including the ports, was taken as the *reference* scenario. For this evaluation, two ROIs were considered: the irradiated breast skin and chest wall volumes (including subcutaneous tissue and underlying muscle), which were created as the intersection of each structure with the 95% isodose (see figure 4).

**Figure 1.**
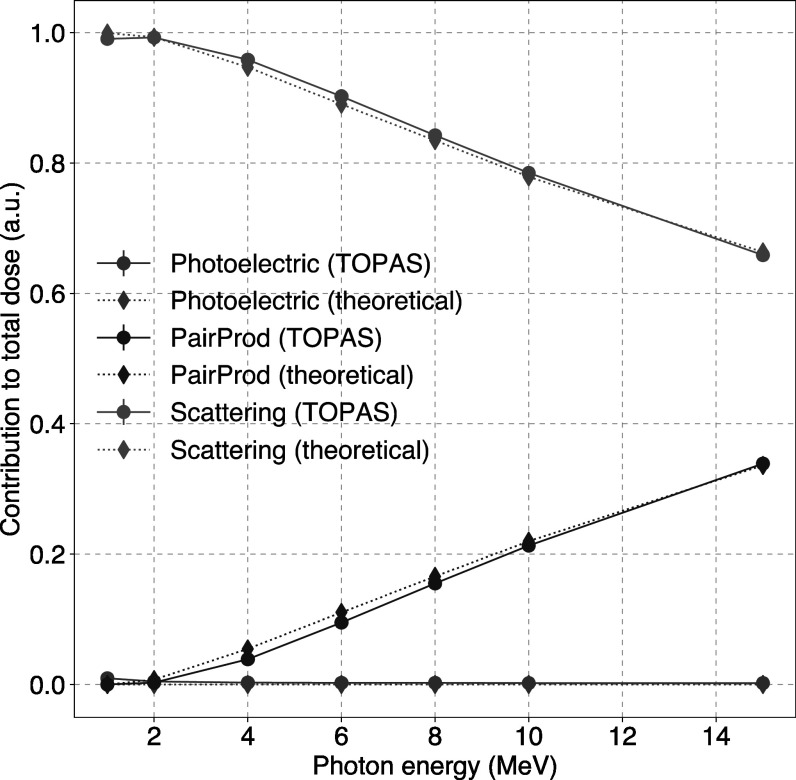
Contribution to the total dose in water from the photoelectric, pair production, and scattering computed by TOPAS and derived from theoretical mass attenuation coefficients (Grodstein, 1957) as a function of the x-ray energy.

For dose calculations, we employed physics parameters recommended for x-ray radiotherapy applications due to their reported balance between accuracy and computational efficiency (Arce *et al* 2020). Specifically, the physics lists consisted of the g4em-standard_opt4 module, and the production cut for the generation of secondary particles was set to 0.5 mm. In the treatment plan including the ports, the contribution to the total dose of different electromagnetic physical processes that may contribute to the dose in normal tissues and organs-at-risk (OARs) were individually assessed. To do so, the interactions within the patient volume that resulted in photon incoherent scattering, photoelectric effect, pair production and bremsstrahlung were not allowed; therefore, the energy deposited from any process but for the one considered was scored in each simulation. An individual simulation was performed with each process deactivated using the *Tsem-standard_opt4* TOPAS physics module. This module allows switching off individual electromagnetic processes. Then, the contribution of each process was calculated by subtracting the scored dose from the dose deposited, considering all processes. To verify the consistency of this implementation, we compared the contribution of these processes to the total dose derived from the theoretical mass attenuation coefficients in water (Grodstein 1957) at relevant clinical x-ray beam energies (1–15 MeV), as illustrated in figure 1. The integral dose computed by TOPAS over a 40x40x40 cm^3^ target volume irradiated by 8 × 8 cm^2^ monoenergetic beams of different energy was considered for this comparison.

### 2.4. Neutron contamination

For neutron production and transportation, the *TsPhotoNeutron* and *g4h_elastic_LEND* modules were used. The *TsPhotoNeutron* module, implemented as an extension to the TOPAS code, was validated against experimental data for the prediction of photoneutron production in high-Z materials in a previous study (Ramos-Mendez *et al* 2024). The yield of neutrons produced by photonuclear reactions in the metallic ports was evaluated by independently scoring the neutrons produced in the patient and simulated component of the linear accelerator (linac) treatment head (i.e., collimation devices). The scoring volume consisted of a spherical shell of 35 cm diameter and 1 cm thickness enclosing the patient. Neutrons created within linac collimation components and patient structures (including TTE) were scored in the outer and inner surfaces of this volume, respectively. The scoring filter by particle’s origin volume of TOPAS was also applied. In addition, the equivalent neutron dose was scored in the patient volume in scenarios with and without the metallic ports. A detailed analysis of the photoneutron production in linac components was out of the scope of this work and can be found in the literature (Becker *et al* 2007).

## 3. Results

### 3.1. Impact of metallic ports on the total dose

**Figure 2.**
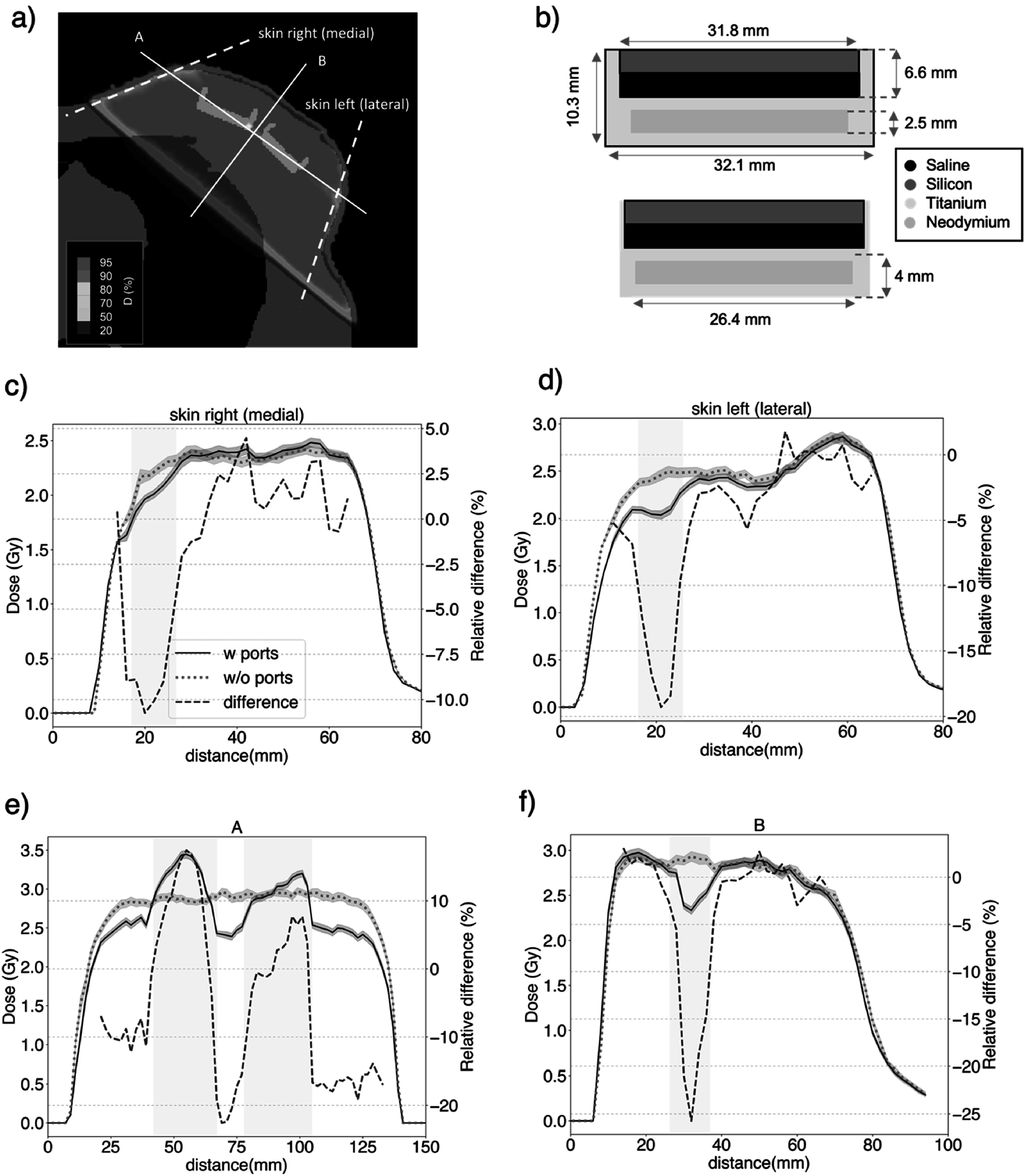
(a) Dose distribution in the treatment plan considering the metallic ports. (b) Geometry of AlloX2 ports (Ramos-Méndez *et al* 2023). Profiles calculated along the lines shown in (a) for the plans with (w ports) and without (w/o ports) ports at the skin on the (c) left and (d) right sides of the patient breast, and within the TTE both in (e) the beam direction and (f) perpendicular to the beam. In all panels, the right *y*-axis corresponds to the relative dose difference in these two scenarios, defined as 100 × (w port - wo port)/w port (%). The position and shadow of the ports in the profiles are represented by a yellow shaded region.

The effect of the metallic ports in dose distribution was evaluated by comparing dose profiles in the scenario with the ports (*w ports*) and without the ports (*w/o ports*) included in the simulation. Four dose profiles were considered in the transverse plane centered on the ports, as shown in figure 2.

**Figure 3.**
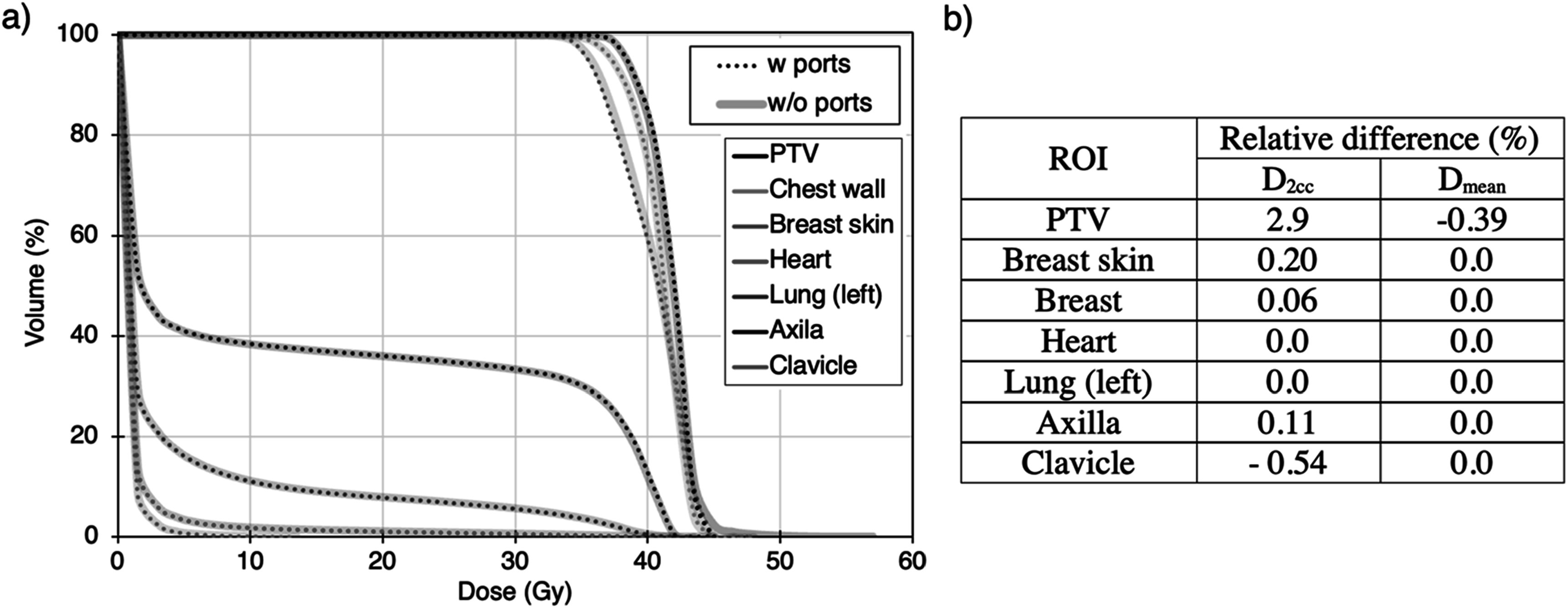
(a) Dose volume histogram of relevant patient structures in the plans with (w ports) and without (w/o ports) metallic ports. (b) Relative difference in the mean and maximum (D_2cc_) dose to relevant patient structures between the plans with and without (reference scenario) metallic ports.

Significant dose differences were observed between these two scenarios, showing that the presence of the ports had a significant dosimetric impact both within the TTE and normal tissue. Distal to the ports, a dose decrease of up to 25.7 ± 0.8% and 19.3 ± 0.6% was observed within the TTE and breast skin, respectively, due to the presence of the ports (see figure 2). No significant dose discrepancies were found in transverse slices outside the projection of the fields through the ports. The dose coverage of the chest wall tissue, assessed by V_95%_ and V_100%_ dosimetric indexes, was not significantly affected (<0.2%) by the presence of the ports (V_95%_ of 92.53% and 92.45%, V_100%_ of 75.67% and 75.56% in the w ports and w/o ports scenarios, respectively). No significant impact (<0.6%) was observed on the mean and maximum (D_2cc_) dose to other relevant patient structures such as the heart, lungs, axilla, or clavicle, as shown in figure 3. A higher difference is found in the maximum dose to the PTV (2.9%) due to the higher dose deposited in the metallic ports, which are included in the PTV.

### 3.2. Contributions of physical processes to the total dose

**Figure 4.**
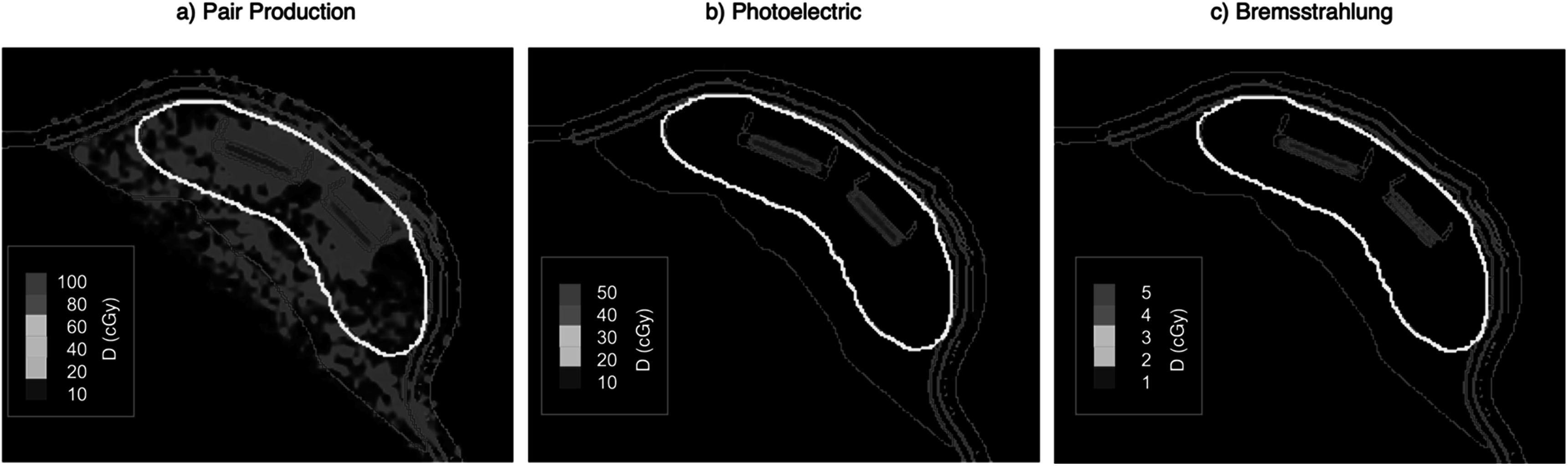
Dose distributions produced by (a) pair production, (b) photoelectric, and (c) Bremsstrahlung processes. The metallic ports are displayed in brown color, the TTE balloon in yellow, the breast skin in blue, the bolus in green, and the chest wall tissue in purple. Doses correspond to doses per fraction (40 Gy in 15 fraction treatment).

Figure 4 shows the dose distributions produced by pair production, photoelectric, and bremsstrahlung processes produced within the patient volume. Contributions from the photoelectric effect and bremsstrahlung are only observed within the metallic ports. The mean dose deposited by these processes in the ports is 9% and 1% of the total dose, respectively. Pair production contributes significantly to the dose to the ports (20% of the total dose) and to the TTE filling (6% of the total dose). The contribution of this process to the breast skin and chest wall was 2.25 Gy and 2.4 Gy in the total treatment course, respectively, amounting to 5.6% and 6% of the prescribed dose. In addition, pair production contributes to the dose deposited in OARs like the left lung and heart; the maximum dose (D_2cc_) to these organs was 2.2 Gy and 0.9 Gy in the whole treatment, respectively, amounting to 5.5% and 2.3% of the prescribed dose. A similar dose to OARs was found when considering the whole breast as water (i.e., not considering the TTE materials), then, the contribution of pair production in this case was not caused by the presence of the TTE, but due to the interaction of high energy x-rays (10 MV) with soft tissues.

### 3.3. Neutron contamination

No neutrons from the patient volume (including the metallic ports) were scored in the 35 cm-diameter enclosing the patient CT, suggesting that it is unlikely that the presence of the TTE will create significant neutron contamination in a 10 MV tangential beam PMRT treatment. Indeed, no significant differences in the equivalent neutron dose to different OARs were observed between the treatment plan dose distributions calculated with or without the ports. Table 1 shows the difference in maximum equivalent neutron dose (H_2cc_) to the OARs considered in both scenarios. The neutron dose in those plans was deposited by neutrons created in the collimation components (jaws and multi-leaf collimator) of the *TrueBeam* system. A further analysis of the photoneutron production in other LINAC components was out of the scope of this work.

**Table 1.** Maximum equivalent neutron dose (H_2cc_) per Gy in plans with and without (reference scenario) metallic ports and their relative difference.

ROI	Maximum equivalent neutron dose per treatment Gy (mSv/Gy)^a^		
	W ports	W/O ports	Difference (%)
Heart	0.287	0.303	−5.3
Lung (left)	0.367	0.373	−1.8
Lung (right)	0.373	0.368	1.5
Axilla	0.125	0.128	−2.1
Clavicle	0.165	0.173	−4.6
Body	0.505	0.508	−0.5

^a^ The statistical uncertainty in the neutron dose calculation was below 0.3%.

## 4. Discussion

Post-mastectomy radiation therapy (PMRT) is typically used to prevent the development of secondary cancers due to the microscopic spread of tumor cells. These treatments may involve temporary tissue expanders (TTE) containing metallic high-Z structures, which may have a significant impact on the dosimetry. In this work, we have studied the dosimetric impact of a dual-port TTE in a clinical case treated with 10 MV tangential beams. This modality and energy may constitute a treatment option in cases involving relatively bulky breasts to improve dose uniformity within the planned target volume and spare skin tissue, compared to 6 MV treatments.

The presence of the metallic ports in the AlloX2 TTE reduces the dose at the proximity of these structures by up to 25%. A less significant but non-negligible reduction was also observed at the skin and normal tissue distal to the ports (by up to 19.3%). These results are consistent with previous studies reporting the dosimetric effect of other single- and dual-port TTE and energies(Thompson & Morgan 2005, Damast *et al* 2006, Chatzigiannis *et al* 2011). For instance, Damast *et al* (Damast *et al* 2006) reported 22% and 16% attenuation in targeted breast tissues for single incident 6 MV beam and 15 MV tangent beams, respectively. Whereas the dose attenuation at the proximity of the ports is not critical for the treatment outcome, since this effect is restricted to the TTE, the dose reduction to the breast skin and normal tissue should be taken into consideration in the treatment planning process since microscopic tumor spread may occur in these structures. Regarding OARs, no significant impact was found in the maximum or mean dose to those structures by the presence of the ports.

The dosimetric impact of the metallic ports within the TTE and patient tissues has been hypothesized to be caused by the different physical processes of energy deposition of high-energy x-rays, such as the photoelectric effect and pair production (Chatzigiannis *et al* 2011). This study investigates those assumptions by explicitly evaluating the individual contributions of these processes to the total dose in the presence of the ports. Bremsstrahlung and photoelectric effects only contribute significantly to the total dose delivered within the metallic ports (1% and 9% of the total dose, respectively). Conversely, pair production contributes 6% of the total dose to the skin and normal tissue, besides its contribution to the ports (11%). The dose deposition by these processes to the dose to the ports may be a potential cause of the dose differences between commercial treatment planning systems (TPS) and predictions of MC simulations (Thompson & Morgan 2005, Gee *et al* 2016). Most clinical TPS use analytical algorithms based on pre-calculated dose kernels that include all electromagnetic photon interactions in a reference material (typically water). However, these kernels are generally scaled by mass density and do not explicitly account for variations in the radiation transport caused by a material of different compositions, which can influence high-energy interactions such as pair production. In this study, pair production contributed 2.2 Gy and 0.9 Gy to the maximum dose to the left lung and heart (see section 3.2), respectively, over the course of treatment (corresponding to 5.5% and 2.3% of the prescribed dose). These contributions appear to result from the use of high-energy (10 MV) x-rays rather than from the presence of the TTE itself. As shown in figure 1, the relative dose contribution from pair production increases with photon energy. While the observed dose to the heart remains below clinical thresholds, the dose produced by this mechanism may be of relevance in scenarios involving cardiac implantable electronic devices (e.g., pacemakers), which have dose limits around 2 Gy (Ohno *et al* 2021). In such cases, modeling pair production with accurate material composition may be needed.

Finally, this work also studies a potential source of neutron contamination in this treatment modality, i.e., the neutrons produced by photoneutron interactions of high-energy x-rays with high-Z materials. As widely discussed in the literature, neutron contamination may potentially contribute to the risk of developing secondary malignancies due to their high biological effectiveness and long ranges in body tissues, allowing the deposition of their energy in organs distant from the primary beam (Expósito *et al* 2013, Seth *et al* 2014). This evaluation was performed using a TOPAS physics list validated against experimental data for the production and transport of photoneutrons (Ramos-Mendez *et al* 2024). The results of this study show that a 10 MV linac x-ray spectrum is not likely to produce photoneutrons in the metallic ports. The equivalent neutron dose is mostly deposited by neutrons produced in the components of the linear accelerator treatment head. Indeed, no significant differences were found in the neutron equivalent dose to OARs between scenarios including and not including the metallic ports. Although the 10 MV x-ray spectrum produces photoneutrons in the collimator system, the dimension of TTE metallic ports is not large enough to interact with the necessary number of photons to create a yield of neutrons contributing to the dose to the patient. These results provide evidence that the presence of metallic ports in 10 MV PMRT treatments may not be a concern for the development of late-side effects compared to treatments without ports. This finding may extrapolate to radiotherapy treatments using lower x-ray energies (e.g., 6 MV) since the probability of photonuclear reactions decreases with the x-ray energy. However, it would be prudent to confirm these results with experimental data.

## 5. Conclusions

This work extends the current knowledge on the impact of the metallic ports of temporary tissue expanders (TTE) on dose distributions in post-mastectomy radiotherapy treatments by studying the individual contribution of the different interactions of x-rays with those structures. Furthermore, it explores the production of photoneutrons in the metallic ports and subsequent dose deposition in organs at risk.

## Data Availability

All data that support the findings of this study are included within the article (and any supplementary files).
